# Clinically Relevant Interactions with Anti-Infectives on Intensive Care Units—A Multicenter Delphi Study

**DOI:** 10.3390/antibiotics10111330

**Published:** 2021-10-31

**Authors:** Joachim Andreas Koeck, Heike Hilgarth, Andreas von Ameln-Mayerhofer, Damaris Meyn, Ruediger Warlich, Andreas Münstedt, Dagmar Horn, Christina König

**Affiliations:** 1 Committee for Intensive Care Medicine and Clinical Nutrition, German Association of Hospital Pharmacists (ADKA), 10559 Berlin, Germany; hilgarth@adka.de (H.H.); a.ameln-mayerhofervon@klinikverbund-suedwest.de (A.v.A.-M.); damaris.meyn@gnh.net (D.M.); ruediger.warlich@sana.de (R.W.); dagmar.horn@ukmuenster.de (D.H.); ch.koenig@uke.de (C.K.); 2 Department of Intensive Care and Intermediate Care, RWTH Aachen University Hospital, 52074 Aachen, Germany; 3 Hospital Pharmacy, RWTH Aachen University Hospital, 52074 Aachen, Germany; 4 Institute of Clinical Pharmacology, RWTH Aachen University Hospital, 52074 Aachen, Germany; 5 Committee for Pharmacists’ Interventions, German Association of Hospital Pharmacists (ADKA), 10559 Berlin, Germany; 6 Hospital Pharmacy, University Medical Center Hamburg-Eppendorf, 20246 Hamburg, Germany; 7 Department of Intensive Care Medicine, University Medical Center Hamburg-Eppendorf, 20246 Hamburg, Germany; 8 Hospital Pharmacy, Sindelfingen-Boeblingen Medical Center, 71065 Sindelfingen, Germany; 9 Hospital Pharmacy, Gesundheit Nordhessen Holding AG, 34121 Kassel, Germany; 10 Hospital Pharmacy, Sana Medical Center Offenbach, 63069 Offenbach, Germany; 11 Hospital Pharmacy, University Hospital Muenster, 48149 Muenster, Germany; andreas.muenstedt@ukmuenster.de

**Keywords:** drug–drug interactions, clinical pharmacy, intensive care unit, critical care pharmacist

## Abstract

Patients in intensive care units (ICUs) are at high risk of drug–drug interactions (DDIs) due to polypharmacy. Little is known about type and frequency of DDIs within German ICUs. Clinical pharmacists’ interventions (PI) recorded in a national database (ADKA-DokuPIK) were filtered for ICU patients. Binary DDIs involving ≥1 anti-infective agent with >1 database entry were selected. A modified two-step Delphi process with a group of senior hospital pharmacists was employed to evaluate selected DDIs for clinical relevance by using a five-point scale and to develop guidance for clinical practice. In total, 16,173 PI were recorded, including 1836 (11%) DDIs in the ICU setting. Of the latter, 41% (756/1836) included ≥1 anti-infective agent, 32% (590/1836) were binary DDIs, and 25% (455/1836) were listed at least twice. This translates into 88 different DDIs, 74% (65/88) of which were rated as being clinically relevant by our expert panel. The majority of DDIs (76% [67/88]) included macrolides, antifungals, or fluoroquinolones. This percentage was even higher in DDIs being rated as clinically relevant by the experts (85% [55/65]). It is noted that an inter-professional discussion and approach is needed in the individual patient management of DDIs. The guidance developed might be a tool for decision support.

## 1. Introduction

Critically ill patients typically receive polypharmacy and are at high risk for drug–drug interactions (DDIs) [1,2,3]. DDIs occur between two or more drugs, which may result in an altered effect or even toxicity of at least one drug. Two recent reviews reported a prevalence of DDIs between 58–67% during ICU stays [4,5]. Due to the patients’ critical condition and continuous monitoring in the ICU, not all DDIs are considered clinically relevant [6]. However, 38% of ICU patients will experience at least one clinically relevant DDI during their ICU stay [6].

Since infections are an important issue in the ICU, a significant number of patients will receive at least one anti-infective agent at some stage during their treatment [7]. In consequence, adequate knowledge of most common interactions between anti-infectives and other drugs and their clinical consequences is necessary [7]. If undetected, DDIs can lead to patient harm or to potentially fatal adverse events [7].

The two main mechanisms known for DDIs are pharmacodynamic and pharmacokinetic alterations. Many of the major pharmacokinetic interactions affect hepatic cytochrome P-450 (CYP) enzymes [8,9]. Interactions involving CYP generally are of two types. The first one is enzyme induction, which leads to an increase of hepatic metabolism, resulting in decreased serum levels of CYP substrates, increasing the risk of therapeutic failure [8,9]. The second type is enzyme inhibition, leading to a reduced metabolism, an increase of serum levels resulting in an increased risk of overdosing and manifestation of potentially fatal adverse events (AE) [8,9].

A frequently recorded pharmacodynamic interaction involves QTc-prolonging drug–drug combinations. In contrast to its high prevalence, only 0.7% of all hospitalized patients in a six-month period developed a severe acquired prolonged long QT-syndrome (ALQTS, i.e., ≥500 ms) [10]. Furthermore, even in the case of an ALQTS, the prevalence of syncope and life-threatening arrhythmia (torsade de pointes) was found to be only 5.8% [10]. Additional risk factors must be considered when assessing the clinical relevance of these DDIs in the individual patient, such as serum potassium and magnesium levels, heart failure, acute myocardial infarction, or acute sepsis. The Tisdale score takes these risk factors into account and has been validated in cardiac and medical ICU patients [11,12]. Thus, it should be applied to daily practice to prevent over-alerting due to QTc-related DDIs.

Serotonin syndrome is known as a potentially life-threatening condition associated with increased central and peripheral serotonergic activity in the central nervous system [13]. It can manifest with therapeutic medication use and especially as a consequence of DDIs [13,14]. Although commonly described as the triad of mental status changes, autonomic hyperactivity, and neuromuscular abnormalities, serotonin syndrome can occur in the absence of an elevated temperature or monoamine oxidase inhibitor treatment, and fast onset cannot be regarded as a reliable clinical sign [13,14].

In order to limit the high number of possible interactions to those which are of clinical significance, our study group used a two-step Delphi process with the aim to describe clinically relevant DDIs involving anti-infective agents, which commonly occur in critically ill patients. In addition, we developed guidance on how to manage these in clinical practice.

## 2. Results

### 2.1. Drug–Drug Interactions from the ADKA-DokuPIK Database

**Figure 1:**
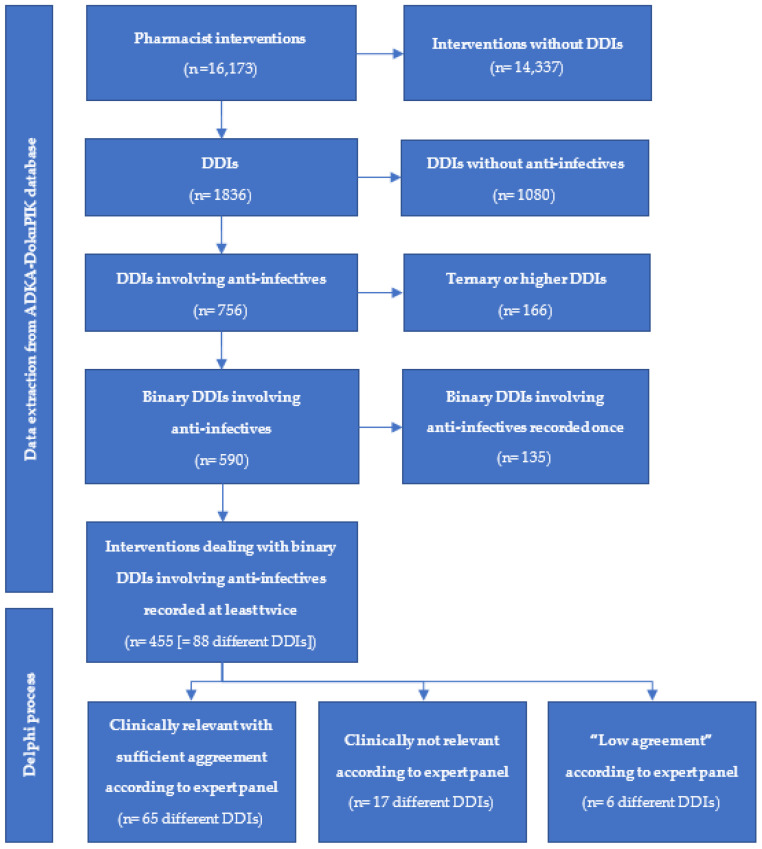
Selection of binary DDIs out of pharmacist’s interventions on German intensive care units recorded in the ADKA-DokuPIK database. DDI = drug–drug interaction.

The German ADKA-DokuPIK database comprised 16,173 PI from ICUs that were recorded over 13.5 years until 2021. Of these, 11% (1836/16,173) described a DDI, of which 41% (756/1836) involved at least one anti-infective agent. A total of 32% (590/1836) were binary drug combinations, with 455 DDIs (455/1836 [25%]) being recorded at least twice (see Figure 1). Out of 455 DDIs, 88 different binary drug–drug combinations were identified (see Table S1).

### 2.2. Ratings of the Expert Panel within the Modified Two-Step Delphi Process

The expert panel comprised of senior clinical pharmacists working on interdisciplinary (6/7 pharmacists), neurology (3/7), and neonatal and pediatric (1/7) ICUs as well as on burns units (1/7), with a professional experience of at least 10 years (IQR 10–16 years).

Within the first Delphi round, consensus was achieved on 59% (52/88) of DDIs, increasing to 93% (82/88) by the end of the second round. Low agreement was attained for 7% (6/88) (Table S3).

In total, 74% of DDIs (65/88) were rated as clinically relevant with sufficient agreement (Table 1 and Table 2). Macrolides (29/88), antifungals (22/88), and fluoroquinolones (16/88) were involved in 76% (67/88) of all DDIs and in 85% (55/65) of those DDIs with clinical relevance according to our expert panel. Acknowledging the initial dataset from the ADKA-DokuPIK database, DDIs rated as clinically relevant frequently included fluoroquinolones (15/16, 94%), antifungals (19/22, 86%), macrolides (21/29, 72%), and rifampicin (4/6, 67%), whereas interactions with, e.g., linezolid were considered less relevant by our expert panel (1/7, 14%). Of all DDIs, 19% (17/88) were rated as not clinically relevant by the authors (see Table S2). Among these, seven were “not relevant at all” and ten “relevant but with low risk for AE due to routine monitoring”. Only for 7% of all DDIs (6/88), the expert panel determined a “low agreement” (see Table S3). The discussion that led to the low agreement is briefly described in Table S3 to provide guidance in daily practice. Of note, all 88 DDIs were initially considered clinically relevant by the pharmacist in the individual patient context that entered the DDI into the database.

For most DDIs (46/65) rated clinically relevant (Category 3), additional monitoring could help to limit toxicities (see Table 2). Nineteen DDIs required therapy modification as they may not be controlled by additional monitoring (Categories 4 and 5).

In total, the expert panel developed 81 recommendations for 65 clinically relevant DDIs. Therapeutic drug monitoring (TDM), electrocardiogram (ECG) monitoring for QTc-prolongation, and monitoring of creatine kinase (CK) or withholding a drug was recommended for 25, 22, and 14 DDIs, respectively. Therapy modification (e.g., switching to an alternative drug) was recommended for seven DDIs.

### 2.3. Interaction Fact Sheets

#### 2.3.1. Cephalosporins

##### Ceftriaxone and Calcium-Containing Intravenous Solutions

Co-administration of intravenous ceftriaxone and **calcium**-containing solutions (i.e., calcium administration for therapeutic purposes or as part of a solvent) may result in precipitation of ceftriaxone–calcium salts. This interaction may also occur when both drugs are administered via Y-site infusion [16]. Therefore, this DDI was rated relevant consistently by all consulted databases.

Ceftriaxone is mainly excreted via renal and biliary pathways. There are pediatric reports showing ceftriaxone–calcium precipitates that resulted in nephrolithiasis [17], biliary sludge or stones [18,19]. In neonates up to an age of 28 days, ceftriaxone is contraindicated according to the summary of product characteristics (SmPC), as precipitation may occur even when ceftriaxone and calcium are administered at different sites [16]. In patients >28 days, ceftriaxone and calcium may be administered at different sites or when infusion lines are sufficiently flushed between administration of these drugs. In case of symptoms for nephrolithiasis or biliary sludge/stones, ultrasound imaging is recommended, and discontinuation of ceftriaxone should be considered.

#### 2.3.2. Carbapenems

##### Carbapenems and Valproic Acid

The interaction of carbapenems and **valproic acid** (VPA) is significant, of rapid onset, and should be avoided if possible [20]. It leads to decreased serum levels of VPA and may result in loss of seizure control and an increase of seizure frequency [21,22]. The exact pharmacokinetic mechanism is poorly understood. Animal studies suggest a reduced intestinal absorption and enterohepatic recirculation [23]. An increase of glucuronidation and a decrease of hepatic hydrolysis resulting in an increased renal clearance of VPA-glucuronide have been postulated [24,25,26,27]. Inhibition of efflux of VPA from erythrocytes and its accumulation have also been described [28,29].

The decrease of VPA levels was highest with meropenem (77%), followed by ertapenem (71%) and imipenem (52%) [30]. Serum levels declined within 24–72 h and were found to be subtherapeutic within 4 days. VPA levels remained low despite VPA dose-increase and were not dependent on meropenem dosages [22,31]. After discontinuation of carbapenem therapy VPA levels returned to the therapeutic range after 8–14 days [30,32]. A case series illustrated that even a short course of meropenem may have long-lasting effects (4 weeks) on VPA serum levels [33].

The concomitant use of carbapenems and VPA should be avoided based on the SmPC and database recommendations. Selection of an alternative anti-infective agent and/or (additive) antiepileptic drug should be discussed based on patients’ individual characteristics (e.g., organ function, microbiology results, seizures frequency/type, or drug history). The additive antiepileptic therapy should be continued for up to 7 days after discontinuation of carbapenem therapy, and VPA serum levels should be checked regularly [32].

#### 2.3.3. Fluoroquinolones

##### Fluoroquinolones and QTc Prolonging Drugs

Fluoroquinolones can prolong the QTc interval, with moxifloxacin posing the greatest risk [34]. Concomitant use of other drugs prolonging the QTc interval such as **class III antiarrhythmics** (e.g., amiodarone), **SSRIs** (e.g., citalopram), **Noradrenaline and specific serotonergic antidepressants** (e.g., mirtazapine), **tricyclic antidepressants** (e.g., amitriptyline), and **antipsychotics** (e.g., haloperidol) can increase the risk of arrhythmias [35,36]. In general, the SmPC advises caution and additional monitoring. Particularly, combining a fluoroquinolone with amiodarone is not recommended by the SmPC, and alternative medication should be discussed.

Despite conflicting database ratings, one can conclude that additional measures such as ECG monitoring are useful to detect prolonged QTc intervals in order to avoid AE. Moreover, prior to the administration of QTc prolonging drug combinations, the screening for risk factors by applying the Tisdale score is reasonable to evaluate the potential threat (see Section 1: Introduction) [11].

##### Fluoroquinolones and Polyvalent Cations or Simvastatin

DDIs with **polyvalent cations** such as iron and calcium with fluoroquinolones do appear frequently when using oral anti-infective therapy and can be avoided by using separate dosing schedules (see Table 2). Moreover, patients using a combination of **ciprofloxacin** (CYP 3A4 inhibitor) and **simvastatin** (CYP 3A4 substrate) should be monitored for tendinopathies and CK-increases (for more details, see Section 2.3.4 Macrolides).

#### 2.3.4. Macrolides

##### Macrolides and CYP 3A4 Substrates

Unlike azithromycin, both clarithromycin and erythromycin inhibit CYP 3A4 enzymes resulting in reduced metabolism of various **statins** (simvastatin and atorvastatin) [37]. Drug exposure is markedly increased, and the risk of myopathy and rhabdomyolysis rises [38]. The combination of CYP 3A4 inhibiting macrolides and statins is usually not recommended by SmPCs. When possible, affected statins should be withheld until the end of the macrolide therapy [39]. If concurrent use is unavoidable, the statin therapy should be switched to another statin where dosing recommendations are available (e.g., atorvastatin) and monitoring of elevated CK and muscle tenderness should be performed. Alternatively, therapy can be changed to a statin, which is not substrate to CYP 3A4 (e.g., rosuvastatin, fluvastatin or pravastatin).

The combination of erythromycin or clarithromycin with **tacrolimus** and/or **cyclosporine** (both CYP 3A4 substrates) will lead to increased concentrations of the immunosuppressants [40]. This may result in potentially toxic serum levels, nephrotoxicity, and prolonged immunosuppression. A combination of CYP 3A4 inhibiting macrolides with immunosuppressants (e.g., tacrolimus and cyclosporine) should be avoided if possible. If concurrent use is unavoidable, immunosuppressant serum levels should be frequently monitored and dosages adjusted accordingly [39].

##### Macrolides and Antidepressants or Antipsychotics

All macrolides are associated with prolongation of the QTc interval and have different cardiac safety profiles. In vitro studies show a variety of causative mechanisms such as formation of reactive oxygen species, block of potassium channels, as well as effects in the cardiomyocyte mitochondria being responsible for their cardiotoxic adverse effects [41,42]. Moreover, macrolide antibiotics showed different potential in causing arrhythmias (erythromycin > clarithromycin > azithromycin) [43]. A recently published meta-analysis evaluated patients receiving erythromycin or clarithromycin being at a higher risk of myocardial infarction (OR = 1.58 and OR = 1.41) when compared with azithromycin [44]. In combination with other QTc prolonging agents such as **antidepressants** and **antipsychotics** (e.g., quetiapine, melperone, haloperidol, or citalopram) the risk of cardiac adverse events such as torsade de points increases. If macrolides are used in combination with other QTc-prolonging agents, the approach as suggested in Section 1: Introduction should be used [11].

#### 2.3.5. Antifungals

##### Echinocandins

Caspofungin undergoes slow metabolic transformation but uses hepatic transporters such as the OATP-1B1 (Organic Anion Transporting Polypeptide) [45]. Therefore, coadministration of caspofungin and **cyclosporine** (substrate of OATP-1B1-transporters) increased the AUC of caspofungin by 35% [46]. In contrast, cyclosporine serum levels were not increased [46,47]. By an unknown mechanism, caspofungin also has the potential to alter **tacrolimus** pharmacokinetics as AUC and C_min_ of tacrolimus were found to be reduced by about 25% [46]. These are preliminary results and still under debate [47]; thus, their clinical relevance is uncertain. A higher exposure of caspofungin appears uncritical due to its low potential for adverse effects. However, regular monitoring of the serum levels of cyclosporine and tacrolimus seems to be reasonable when using caspofungin.

##### Azole Antifungals

The azole antifungals itraconazole, fluconazole, posaconazole, voriconazole, and isavuconazole are frequent partners in clinically relevant DDIs due to their inhibition of fungal and human CYP enzyme systems (see Table 3) [48].

A combination of azoles causing moderate to strong inhibition of CYP 3A4 with **statins** such as atorvastatin and simvastatin (CYP 3A4 substrates) is usually not recommended (SmPC) due to an increased risk of statin toxicity. Clinical management is described in the Section 2.3.4. Macrolides.

A combination of **immunosuppressants** such as cyclosporine or tacrolimus with CYP inhibiting azoles results in a marked increase of drug exposure of the immunosuppressants with potentially toxic effects [49,50,51]. Hence, a concurrent use of azoles and cyclosporine or tacrolimus should be avoided. If unavoidable, an initial dose reduction of the immunosuppressants by about 30–50% is recommended with further therapy being guided by TDM (SmPC). A similar approach applies to **sildenafil** (CYP 3A4 substrate) as azoles increase its serum level 10-fold, leading to a risk of AE (e.g., excessive blood pressure-lowering) [52]. Therefore, concurrent administration of an azole is not recommended by the SmPC, and alternative antifungal agents such as echinocandins or liposomal amphotericin B should be discussed.

Concurrent use of azoles (e.g., posaconazole or fluconazole) with **carbamazepine** (CYP 3A4 substrate and strong inducer) results in altered pharmacokinetics of both substances. Inhibition of carbamazepine metabolism increases its serum level up to 140%, resulting in a high risk for AE. On the other hand, carbamazepine co-administration can result in decreased azole serum levels (e.g., voriconazole), increasing the risk for therapeutic failure. Close monitoring for increased carbamazepine levels and toxicity (e.g., ataxia, drowsiness, and vertigo) as well as azole levels is highly recommended.

The use of azoles (CYP substrate, e.g., voriconazole and isavuconazole) in **rifampicin** (CYP 3A4 inducer)-treated patients leads to an inadequate exposure and increased risk of failure of the antifungal therapy. The SmPC recommends avoiding this combination. When antifungal therapy cannot be switched to alternatives (e.g., echinocandins or liposomal amphotericin B), a suitable alternative for rifampicin should be discussed such as fosfomycin in the case of biofilm-producing microorganisms [53]. Alternatively, azole TDM can be performed especially in patients with extra-corporeal organ support and with regard of reduced azole exposition due to DDI [54]. If TDM is not available, SmPC for **fluconazole** recommends a dose increase of 25% in combination with rifampicin.

The oral liquid of posaconazole shows a highly variable bioavailability especially when administered on an empty stomach, indicating pH dependent absorption. By increasing gastric pH, e.g., by **proton-pump inhibitors** (PPI), absorption of posaconazole suspension is markedly reduced [55]. To avoid therapeutic failure, concurrent use of a PPI should be avoided or posaconazole administered as a modified-release tablet (when swallowing is possible) [56] or as an intravenous infusion.

#### 2.3.6. Miscellaneous Anti-Infectives

##### Daptomycin

Both **statins** and daptomycin can cause an increase in CK and are independently associated with myopathy and rhabdomyolysis [57]. When used concurrently, the SmPC recommends twice-weekly CK-monitoring and consideration of temporarily withholding statins; in the case of a CK increase to five times its normal value, both the statin and daptomycin should be stopped. Additional risk factors (e.g., age > 65 years, female sex, and untreated hypothyroidism) for myopathy and rhabdomyolysis should also be considered. Because of high inter-individual serum level ranges of daptomycin, TDM can help to achieve effective serum levels whilst minimizing the risk of toxicity [58,59].

##### Rifampicin

Rifampicin is a strong inducer for CYP 3A4 and P-gp. Thereby, the concomitant administration can result in altered metabolism or transportation. During concurrent use of **cyclosporine** (CYP 3A4, P-gp substrate) and rifampicin, serum levels of cyclosporine can decrease to subtherapeutic levels, increasing the risk of therapeutic failure. If an alternative immunosuppressant therapy is ineligible, cyclosporine concentrations should be closely monitored. A two- to threefold increase in doses of cyclosporine can be required in these situations [60].

After five days of concurrent use with rifampicin, C_max_ and AUC of **simvastatin** dropped by about 90% and 87% in healthy adults [61]. The SmPC recommends avoiding concurrent use of simvastatin and rifampicin. When statin therapy is unavoidable, a statin that is not substrate to CYP 3A4 (e.g., fluvastatin and pravastatin) may be used.

The oral P2Y12 receptor antagonist **ticagrelor** is substrate to CYP 3A4, and both the parent compound and its active metabolite are substrates of P-gp. Concurrent administration significantly decreased C_max_ by 73%, AUC by 86%, and the serum half-life of ticagrelor by 67%. Moreover, the active metabolite decreased by 46% [62]. Therefore, concurrent use can result in a marked decline of platelet inhibition and should be avoided.

The prodrug **clopidogrel** is primarily metabolized by CYP 2C19 into its active metabolite. In combination therapy with rifampicin, Cmax and AUC of the active metabolite were increased up to fourfold in healthy adults, resulting in an increased platelet inhibition [63]. Because of the high risk for bleeding complications, this combination should be monitored closely (SmPC) or if possible avoided. For both ticagrelor and clopidogrel, prasugrel (minor substrate of CYP 3A4) may be an alternative as rifampicin does not significantly alter its metabolism [64]. For information on DDIs between rifampicin and **azole** antifungals, see Section 2.3.5 Antifungals.

##### Linezolid and Serotonergic Agents

Linezolid was originally investigated as a psychotropic agent with antidepressant effects through mild reversible nonselective inhibition of monoamine oxidase (MAO). Moreover, it was also found to have anti-infective properties against drug-resistant gram-positive cocci [65]. While some case reports showed the occurrence of a serotonin syndrome solely with **mirtazapine** [66,67], there is one case where serotonin syndrome developed under the concomitant therapy of linezolid, mirtazapine, and citalopram [68]. Therefore, a combination of serotonergic agents and linezolid should be avoided. On the other hand, the mediQ^®^ database and Fong et al. estimated the risk of serotonin syndrome with mirtazapine as low, because of its antagonistic impact on 5-HT2-receptors, which might provide a protective effect [69]. If strictly indicated, linezolid may be used in mirtazapine-treated patients, provided there is close monitoring for symptoms of serotonin syndrome (e.g., perspiration, fever, and tachycardia) (see Section 1: Introduction).

As there is a high inter- and intra-individual range of linezolid serum levels and an evident risk of dose-dependent AE, dosing of linezolid should be guided by TDM where available [70].

## 3. Discussion

To the best of our knowledge, this is the first publication to review and interpret clinically relevant DDIs occurring regularly in ICU patients in Germany. PIs, documented in a national database, described regular clinical pharmacists’ activity and input on patient and drug safety. Monitoring and managing DDIs is only one aspect of clinical pharmacy practice on German ICUs. However, clinical pharmacists are an essential team member when detecting, evaluating, and managing DDIs according to clinical relevance. Considering an individual patient’s condition and requirements, they support other health care practitioners by recommendations on monitoring or suggesting therapeutic alternatives [71].

This study has several strengths. Firstly, by using the previously described ADKA DokuPik^®^ database [72], we were able to describe the natural heterogeneity of critically ill patients. We were able to include PI from a wide range of pharmacists throughout the country. Therefore, we do present a high diversity of DDIs within the medical therapy of critically ill patients with various comorbidities within several ICU types. Secondly, we identified the main anti-infective agents accountable for a considerable number of DDIs in ICUs.

Among all substances that have been recorded in the database three substance classes, namely macrolides, antifungals, and fluoroquinolones were involved in about 76% of all documented DDIs and in 85% of all by our experts clinically relevant rated DDIs. This is in line with several other studies that investigated relevant DDIs in critically ill patients where the same substance classes have been reported as being highly relevant or frequently causing adverse effects [4,6,73]. This parallelism in findings may be due to the nature of the interactions, i.e., the P-gp and/or CYP interaction potential [74,75]. With the essential knowledge of these three groups, the physicians and clinical pharmacists can deal with the most frequent DDIs.

Modern patient data management systems (PDMS) and electronic health records do provide automated DDI checks, often resulting in a flood of drug-associated warnings. One of the fundamental roles of a clinical pharmacist is to become a gatekeeper in the over-alerting of DDI warnings. Clinical pharmacists can support the physicians in benefit-risk assessments, when strictly required polypharmacy raises both the risks for therapeutic failure and potentially fatal AE. Therefore, we have developed guidance on how to manage these DDIs in clinical practice based on SmPC, currently available literature, and DDI databases. It should be particularly emphasized that clinical decision-making is a process requiring interdisciplinary collaboration. With regard to the urgent needs and problems of ICU patients, DDIs must be discussed with the treating physicians. In this context, as reported previously by Tisdale et al., risk factors such as QTc prolongation should be assessed in order to evaluate and minimize the occurrence of severe adverse events [11]. Managing CYP interactions is usually well-described in SmPC as well as in clinical studies. However, when clinical or patient data are not available (e.g., in the case of new drugs), in vitro or in silico estimations based on the metabolism and extent of CYP inhibition/induction are a common approach to estimate the effect [76,77].

Last, our clinical relevance definition was based on a Delphi process assessed by a senior expert panel. This method has been described and carried out before [15].

A significant limitation of this work is that the number of pharmacists working on German ICUs is not known. Data suggests that 16.8% of all PI entered in DokuPIK^®^ are generated on ICU/intermediate care units or operating theatres [78]. As the data entry in DokuPIK^®^ is voluntary, data might have been entered by more engaged pharmacists and therefore the results might not be fully applicable to ‘clinical pharmacy practice in Germany’ as a whole. However, the extracted binary DDIs are suitable to provide reasonable and sufficiently reliable results. Within a duration of more than 13 years, only 16,173 PI were entered, which could be due to time restraints and lack of legal requirements for clinical pharmacists to document PI as well as small numbers of registered users. However, 11% of PI were logged as DDI. Hence, this represents only a small proportion of a pharmacist’s daily work.

It must be noted that we only included binary DDIs. DDIs between three or more drugs were excluded as evidence for relevance, and guidance is sparse. Moreover, we did not include all possible DDIs but only those that were included in the ADKA DokuPIK^®^ database. There might be clinically relevant DDIs that we did not include using this method.

At last, the expert panel was formed by senior hospital pharmacists regularly working in ICU but with no further specialist qualification for critical care pharmacy, because there is no structured training in this regard in Germany. We did not involve other groups of health care professionals. Additionally, the senior pharmacists rated the DDIs according to their individual opinion, based on their expertise in the field. This may have influenced the assessment of a DDI being clinically relevant. However, the Delphi process aimed at minimizing this effect, in which an anonymized rating of DDIs by multiple experts took place.

Patients in critical care are especially vulnerable due to life-threatening illness, complex pharmacotherapy, and medical care requirements, in addition to extensive monitoring including diagnostic and laboratory data. In the case of clinically relevant DDIs, the multidisciplinary team will responsibly decide whether monitoring is feasible, and drug-combinations may be used in these special circumstances and comply with local legal requirements.

## 4. Materials and Methods

The national anonymous self-reported online documentation system DokuPIK^®^, hosted by the German Association of Hospital Pharmacists (ADKA), was used for data acquisition. In DokuPIK^®^, pharmacists voluntarily document medication errors (ME) and PIs describing type of intervention, reasons, actions taken, and outcomes. A more detailed description of the DokuPIK^®^ database has been published previously [79].

From DokuPIK^®^, we extracted data for PI entered as binary DDI (interactions between only two drugs) in the ICU setting from 2007 until 2021. Using Microsoft Excel^®^ (Redmond, Washington), binary interactions with at least one anti-infective agent (ATC code “J”—anti-infectives for systemic use [80]) were selected. Combinations of more than two substances were excluded. DDIs had to be listed at least twice to be included in the two-step Delphi process.

### 4.1. Expert Panel and Modified Two-Step Delphi Process

The resulting list of DDIs was assessed by a seven-member expert panel of the ADKA committee for intensive care medicine and clinical nutrition using a modified two-step Delphi process [81]. Rating of clinical relevance was based on five mode categories as reported by Bakker et al. [15]:

- Mode category 1: “Not clinically relevant, since adverse effects of this DDI are negligible”.
- Mode category 2: “Clinically relevant; the adverse effects of this DDI will, however, be limited since routine monitoring to timely identify adverse effects is present”.
- Mode category 3: “Clinically relevant; the adverse effects of this DDI can, however, be limited by additional monitoring and/or changes in dosage/frequency/timing”.
- Mode category 4: “Clinically relevant, the adverse effects of this DDI on the patient can be substantial; however, these effects are acceptable and treatable”.
- Mode category 5: “Clinically relevant; the adverse effect of this DDI on the patient should preferably be avoided”.

A clinically relevant DDI required the majority of experts to decide for Mode Category 3–5 and excluded low agreement. Low agreement was defined in line with Bakker et al. by at least one rating in Mode Categories 1–2 and 5 [15]. If a DDI was scored in Mode Category 3, it was required to provide additional information on preventing or monitoring AE.

For the recommendation and guidance, experts reviewed current literature and SmPCs and consulted six international DDI databases (Medscape^®^ drug interaction checker (United States); Lexicomp^®^ Drug Interactions (Netherlands); Stockely’s Drug Interactions^®^ (Great Britain); mediQ^®^ (Switzerland); AiDKlinik^®^ (Germany); ID-Pharma^®^ (Germany)).

Finishing the first round, all DDIs with low agreement (criteria see above) were extracted and included in the second round of the Delphi process via video conferencing. Those selected were discussed, and the rating was confirmed or revised. In both steps of the Delphi process, confidentiality of expert’s votes was provided to avoid influencing each other.

Some DDIs are referred to as contraindication by manufactures. For these, a multidisciplinary discussion and individual risk–benefit evaluation and documentation were recommended. It was suggested by our expert panel that the following questions should be used to support the multidisciplinary dialog:

1. Are both drugs indispensable for the individual patient?
2. Is there a therapeutic alternative available for one of the drugs?
3. Is there any feasible monitoring available to early identify AE?

### 4.2. Interaction Fact Sheets

To guide clinical management of frequently identified relevant DDIs, a detailed review of mechanism, toxicities, management, and monitoring was prepared.

### 4.3. Data Analysis

The ratings for clinical relevance were ordinal scaled. Therefore, the group ratings were determined via mode. The guidance on preventing or monitoring AE, listed for DDIs in Category 3, was iteratively assigned to five categories. Metric data were displayed with percentages or median and interquartile range, whatever was applicable.

## 5. Conclusions

Clinically relevant DDIs do appear as a result of polypharmacy in ICU. Especially macrolides, antifungals, and fluoroquinolones are often involved in clinically relevant DDIs and require special attention. However, many clinically relevant DDIs can be avoided by choosing an alternative agent. Others can be attenuated by additional or precautionary monitoring such as TDM or ECG. In addition, regular surveillance of DDIs by a clinical pharmacist as part of the multidisciplinary team in the ICU facilitates other healthcare professionals to manage DDIs and can therefore substantially support patient and drug safety.

## Figures and Tables

**Table 1:** Ratings of the expert panel.

Group	Clinically Relevant (n = 65)	Not Clinically Relevant or Low Agreement (n = 23)	Total (n = 88)
Penicillins	0	1	1
Cephalosporins	1	1	2
Carbapenems	2	0	2
Fluoroquinolones	15	1	16
Macrolides	21	8	29
Glycopeptides	0	1	1
Antifungals	19	3	22
Miscellaneous	7	8	15

**Table 2:** DDIs (n = 65) including anti-infectives and rated clinically relevant by expert panel.

Group	Drug 1	Drug 2	Mode Category	Additional Strategies to Reduce Patient Risk from Interaction
Cephalosporins	Ceftriaxone	Calcium (intravenous)	3	separate administration
Carbapenems	Imipenem	Valproic acid	5	TDM for valproic acid
	Meropenem	Valproic acid	5	
Fluoroquinolones	Ciprofloxacin	Calcium	3	separate administration by two hours, or use different administration routes (oral/intravenous)
		Amiodarone	3	QTc-monitoring, high-normal serum levels of potassium and magnesium
		Mirtazapine	3	
		Amitriptyline	3	
		Haloperidol	3	
		Theophylline	3	QTc-monitoring, high-normal serum levels of potassium and magnesium, TDM for theophylline, use 60% of regular theophylline dose
		Iron	3	separate administration by two hours, or use different administration routes (oral/intravenous)
		Melatonin	3	monitor for sleepiness
		Simvastatin	3	CK-monitoring, monitor for signs of myalgia
	Levofloxacin	Amiodarone	3	QTc-monitoring, high-normal serum levels of potassium and magnesium
	Levofloxacin	Haloperidol	3	
	Moxifloxacin	Amiodarone	3	
		Mirtazapine	3	
		Prednisolone	4	monitor for signs of tendinopathy
Macrolides	Azithromycin	Citalopram	3	QTc-monitoring, high-normal serum levels of potassium and magnesium
		Haloperidol	3	
	Clarithromycin	Simvastatin	5	withhold/switch statin
		Atorvastatin	5	CK-monitoring, monitor for signs of myalgia, withhold/switch statin
		Tacrolimus	3	TDM for tacrolimus
		Cyclosporine	3	TDM for cyclosporine
		Carbamazepine	3	where appropriate: switch clarithromycin to azithromycin, TDM for carbamazepine
		Amiodarone	3	QTc-monitoring, high-normal serum levels of potassium and magnesium
		Haloperidol	3	
		Ivabradine	5	QTc-monitoring, high-normal serum levels of potassium and magnesium, monitor for bradycardia
		Moxifloxacin	3	QTc-monitoring, high-normal serum levels of potassium and magnesium
		Theophylline	3	QTc-monitoring, high-normal serum levels of potassium and magnesium, TDM for theophylline
	Erythromycin	Simvastatin	5	withhold/switch statin
		Atorvastatin	5	CK-monitoring, monitor for signs of myalgia, withhold/switch statin
		Quetiapine	3	QTc-monitoring, high-normal serum levels of potassium and magnesium
		Tacrolimus	3	TDM for Tacrolimus
		Amiodaron	3	QTc-monitoring, high-normal serum levels of potassium and magnesium
		Carbamazepine	5	where appropriate: switch erythromycin to azithromycin, TDM for carbamazepine
		Cyclosporine	3	where appropriate: switch erythromycin to azithromycin, TDM for cyclosporine
	Erythromycin	Haloperidol	3	QTc-monitoring, high-normal serum levels of potassium and magnesium, where appropriate: switch erythromycin to azithromycin
		Melperon	3	QTc-monitoring, high-normal serum levels of potassium and magnesium
Antifungals	Caspofungin	Cyclosporine	3	TDM for cyclosporine
	Fluconazole	Cyclosporine	3	
		Tacrolimus	3	TDM for tacrolimus
		Simvastatin	5	CK-monitoring, monitor for signs of myalgia, withhold/switch statin
		Atorvastatin	3	
		Carbamazepine	5	TDM for carbamazepine
		Rifampicin	3	discuss alternatives
	Itraconazole	Simvastatin	5	withhold/switch statin
	Posaconazole	Pantoprazol	3	intravenous administration of posaconazole, TDM for posaconazole
		Cyclosporine	3	TDM posaconazole and cyclosporine
		Atorvastatin	5	withhold/switch statin
		Carbamazepine	3	TDM carbamazepine
		Tacrolimus	3	TDM posaconazole and tacrolimus
	Voriconazole	Tacrolimus	3	TDM voriconazole and tacrolimus
		Simvastatin	5	withhold/switch statin
		Atorvastatin	5	
		Cyclosporine	3	TDM for voriconazole and cyclosporine
		Rifampicin	5	TDM for voriconazole, discuss alternatives
		Sildenafil	3	dose reduction of sildenafil
Miscellaneous	Daptomycin	Atorvastatin	3	CK-monitoring, monitor for signs of myalgia, withhold/switch statin
		Simvastatin	3	
	Linezolid	Mirtazapine	3	monitor for signs of serotonin syndrome
	Rifampicin	Cyclosporine	3	TDM for cyclosporine
		Clopidogrel	5	monitor for signs of bleeding, discuss alternatives
		Simvastatin	5	withhold/switch statin
		Ticagrelor	5	discuss alternatives

**Mode Category 3**: “Clinically relevant, the adverse effects of this DDI can however be limited by additional monitoring and/or changes in dosage/frequency/timing.” **Mode Category 4**: “Clinically relevant, the adverse effects of this DDI on the patient can be substantial, however these effects are acceptable and treatable.” **Mode Category 5:** “Clinically relevant, the adverse effect of this DDI on the patient should preferably be avoided.” [15]. CK = creatine kinase; TDM = therapeutic drug monitoring; QTc = Rate-corrected QT interval.

**Table 3:** Metabolism and CYP inhibition of currently marketed azoles.

Azole	Weak Inhibition	Moderate Inhibition	Strong Inhibition	Substrate
Itraconazole			CYP 3A4	CYP3A4
Fluconazole		CYP 2C9 CYP 3A4	CYP 2C19	
Voriconazole	CYP 2C9	CYP 2C19	CYP 3A4	CYP 3A4 CYP 2C19 CYP 2C9
Posaconazole			CYP 3A4	P-gp
Isavuconazole		CYP 3A4		CYP 3A4

CYP = Cytochrome P-450; P-gp = P-glycoprotein.

## Data Availability

The data presented in this study are available on request from the corresponding author.

## Supplementary Materials

The following are available online at https://www.mdpi.com/article/10.3390/antibiotics10111330/s1, Table S1: Number of 88 different binary drug–drug interactions listed in ADKA-DokuPIK database at least twice and contained at least one anti-infective agent (total n = 455 interactions). Table S2: DDI (n = 17) including anti-infectives rated not clinically relevant by expert panel. Table S3: DDI (n = 6) containing anti-infectives resulting in low agreement of the expert panel.
