# Supplementary material for: Clinically Relevant Interactions with Anti-Infectives on Intensive Care Units—A Multicenter Delphi Study
Source: Antibiotics (Basel). 2021 Oct 31;10(11):1330. doi: 10.3390/antibiotics10111330 (PMC8614667; doi:10.3390/antibiotics10111330)
Supplement: Supplementary file 1 [file antibiotics-10-01330-s001.zip › antibiotics-1424269-supplementary.pdf]

Table S1: Tally of 88 different binary drug-drug interactions (DDIs) listed in the ADKA-DokuPIK database at least twice and including at least one anti-infective agent (total n= 455 interactions)

Drug 1	Drug 2	n
Clarithromycin	Simvastatin	43
Meropenem	Valproic acid	37
Erythromycin	Simvastatin	25
Voriconazole	Tacrolimus	21
Ciprofloxacin	Calcium	15
Clarithromycin	Tacrolimus	15
Voriconazole	Simvastatin	13
Fluconazole	Tacrolimus	12
Ciprofloxacin	Amiodarone	10
Erythromycin	Atorvastatin	9
Posaconazole	Pantoprazole	9
Caspofungin	Cyclosporine	8
Voriconazole	Cyclosporine	8
Voriconazole	Rifampicin	8
Clarithromycin	Cyclosporine	7
Ciprofloxacin	Citalopram	6
Ciprofloxacin	Mirtazapine	6
Linezolid	Norepinephrine	6
Clarithromycin	Atorvastatin	5
Clarithromycin	Carbamazepine	5
Erythromycin	Quetiapine	5
Fluconazole	Simvastatin	5
Imipenem	Valproic acid	5
Linezolid	Citalopram	5
Linezolid	Mirtazapine	5
Posaconazole	Ranitidine	5
Azithromycin	Atorvastatin	4
Erythromycin	Sufentanil	4
Erythromycin	Tacrolimus	4
Linezolid	Piritramide	4
Moxifloxacin	Amiodarone	4
Posaconazole	Cyclosporine	4
Ceftriaxone	Calcium	3
Ciprofloxacin	Amitriptyline	3
Ciprofloxacin	Haloperidol	3
Ciprofloxacin	Theophylline	3
Clarithromycin	Amiodarone	3
Clarithromycin	Fluvastatin	3
Clarithromycin	Haloperidol	3
Clarithromycin	Ivabradine	3
Clarithromycin	Moxifloxacin	3
Clarithromycin	Theophylline	3

Erythromycin	Mirtazapine	3
Fluconazole	Cyclosporine	3
Levofloxacin	Amiodarone	3
Linezolid	Fentanyl	3
Moxifloxacin	Mirtazapine	3
Moxifloxacin	Prednisolone	3
Rifampicin	Fluvastatin	3
Rifampicin	Paracetamol (Acetaminophen)	3
Teicoplanin	Furosemide	3
Voriconazole	Atorvastatin	3
Voriconazole	Clopidogrel	3
Ampicillin-sulbactam	Allopurinol	2
Azithromycin	Citalopram	2
Azithromycin	Haloperidol	2
Caspofungin	Albumin	2
Ceftriaxon	Heparin	2
Ciprofloxacin	Iron	2
Ciprofloxacin	Melatonin	2
Ciprofloxacin	Simvastatin	2
Clarithromycin	Amlodipine	2
Clarithromycin	Trimethoprim-sulfamethoxazole	2
Clarithromycin	Tamsulosin	2
Daptomycin	Atorvastatin	2
Daptomycin	Simvastatin	2
Erythromycin	Amiodarone	2
Erythromycin	Carbamazepine	2
Erythromycin	Cyclosporine	2
Erythromycin	Haloperidol	2
Erythromycin	Melperone	2
Erythromycin	Midazolam	2
Fluconazole	Atorvastatin	2
Fluconazole	Carbamazepine	2
Itraconazole	Simvastatin	2
Levofloxacin	Haloperidol	2
Linezolid	Moclobemide	2
Linezolid	Sufentanil	2
Moxifloxacin	Citalopram	2
Posaconazole	Atorvastatin	2
Posaconazole	Carbamazepine	2
Posaconazole	Tacrolimus	2
Rifampicin	Cyclosporine	2
Rifampicin	Clopidogrel	2
Rifampicin	Fluconazole	2
Rifampicin	Simvastatin	2
Rifampicin	Ticagrelor	2
Voriconazole	Sildenafil	2

Table S2: DDI (n= 17) including anti-infectives rated not clinically relevant by expert panel

Group	Drug 1	Drug 2	Mode category[1]
Penicillins	Ampicillin-sulbactam	Allopurinol	2
Cephalosporins	Ceftriaxone	Heparin	1
Macrolides	Azithromycin	Atorvastatin	2
	Clarithromycin	Fluvastatin	1
		Amlodipine	2
		Trimethoprim-sulfamethoxazole	1
	Erythromycin	Sufentanil	2
		Mirtazapine	1
		Midazolam	2
Glycopeptides	Teicoplanin	Furosemide	1
Antifungals	Caspofungin	Albumin	1
Miscellaneous	Linezolid	Norepinephrine	2
		Citalopram	2
		Piritramide	2
		Fentanyl	2
		Sufentanil	2
	Rifampicin	Fluvastatin	1

Mode category 1: "Not clinical relevant, since adverse effects of this DDI are negligible." **Mode category 2**: "Clinically relevant, the adverse effects of this DDI will however be limited since routine monitoring to timely identify adverse effects is present" [15].

Table S3: DDIs (n= 6) including anti-infectives resulting in low agreement of the expert panel

Group	Drug 1	Drug 2	Discussion that led to low agreement
Fluoroquinolones	Ciprofloxacin	Citalopram	QTc-Monitoring is handled differently in German ICU either as routine or as additional monitoring procedure; SmPC citalopram: CI with ciprofloxacin
Macrolides	Clarithromycin	Tamsulosin	Usually in ICU tamsulosin is withheld when a urinary bladder catheter is placed, in which case the interaction is irrelevant. However, when tamsulosin is continued, a possible hypotension was acknowledged.
Antifungals	Posaconazole	Ranitidine/ Famotidine	If both drugs are given orally, Posaconazole levels might be decreased. However, Posaconazole is often administered intravenously and TDM of Posaconazole often in place.
	Voriconazole	Clopidogrel	Clinical relevance of this interaction was questioned especially in long-term clopidogrel therapy. Testing of platelet-function may be considered.
Miscellaneous	Linezolid	Moclobemide	Serotonine syndrome is a rare but severe adverse drug reaction, so monitoring is recommended; SmPC linezolid: CI with moclobemide.
	Rifampicin	Paracetamol (Acetaminophen)	Clinical relevance for ICU in short-term combination is questioned. Routine monitoring of liver function is recommended. Long-term combination therapy should be avoided, especially without frequent monitoring of liver function.

CI= contraindication; QTc= Rate-corrected QT interval; SmPC= summary of product characteristics; TDM= Therapeutic Drug Monitoring
